# Adsorption Kinetics of Arsenic (V) on Nanoscale Zero-Valent Iron Supported by Activated Carbon

**DOI:** 10.3390/nano10091791

**Published:** 2020-09-09

**Authors:** Huijie Zhu, Mingyan Shi, Xiuji Zhang, Bo Liu, Dahu Yao

**Affiliations:** 1 Henan International Joint Laboratory of New Civil Engineering Structure, College of Civil Engineering, Luoyang Institute of Science and Technology, Luoyang 471023, China; huijiezhu@lit.edu.cn (H.Z.); zhangxiuji@lit.edu.cn (X.Z.); 2 College of Civil Engineering, Guangzhou University, Guangzhou 510006, China; shmygz@gzhu.edu.cn; 3 Laboratory of Functional Molecular and Materials, School of Physics and Optoelectronic Engineering, Shandong University of Technology, Zibo 255000, China; 4 School of Chemical Engineering & Pharmaceutics, Henan University of Science & Technology, Luoyang 471003, China; dh.yao@haust.edu.cn

**Keywords:** nanoscale zero-valent iron supported on activated carbon (NZVI/AC), arsenate (As(V)), adsorption kinetics, intra-particle diffusion

## Abstract

The presence of arsenic (As) in drinking water is of serious concern due to its negative impact on human health. This work reports on the kinetics of nanoscale zero-valent iron (Fe^0^) supported by activated carbon (NZVI/AC) for the removal of As (V) species from aqueous solutions. To better understand the factors affecting this process, we investigated the effects of various experimental parameters including initial As (V) concentration, adsorbent dosage, pH, temperature, and coexisting ions on the adsorption kinetics using a batch-adsorption method. The optimum conditions for As (V) removal by NZVI/AC were found to be: 318 K, pH 3.5, an adsorbent dosage of 1.5 g/L, and an equilibrium time of 72 h. A greater mass of NZVI/AC, lower concentration of As (V) and lower pH positively promoted adsorption kinetics. The presence of phosphate (${\mathrm{PO}}_{4}^{3-}$) and silicate (${\mathrm{SiO}}_{4}^{2-}$) markedly inhibited As (V) removal kinetics. However, in the presence of 4.5 g/L NZVI/AC, ≥99.9% of As (V) was removed from raw groundwater.

## 1. Introduction

Arsenic (As) is associated with serious environmental concerns because of its noted negative impact on human health ranging from acute lethality to carcinogenicity and chronic health effects [1]. Drinking water is one of the major sources of As exposure in the general population, especially in some developing countries, such as Bangladesh, China, and Vietnam. Among various As removal techniques, the zero-valent iron (Fe^0^) adsorption method has been widely adopted because it is simple and cost-effective [2,3,4,5,6,7,8,9,10].

Nanoscale Fe^0^ (NZVI) is a very promising adsorbent for As elimination from drinking water since it possesses a high As adsorption capacity and a large specific surface area. Moreover, NZVI has an As removal mechanism similar to ZVI [11,12,13]. However, the direct application of NZVI in water cleaning systems might result in NZVI losses and secondary contamination of the drinking water. Instead, NZVI supported by activated carbon (NZVI/AC) can safely and effectively remove arsenic due to its great porosity, low cost, and mechanical stability [12,14].

Adsorption kinetics analysis typically provides data on how experimental conditions affect the adsorption speed and rate as a function of time [15]. The solute uptake rate, needed to determine the residence time, is required to assess the adsorption reaction completion. Moreover, the scaling-up of the adsorption equipment also requires kinetic data [16,17].

The kinetics of the removal of As from aqueous solutions by ZVI media has been the subject of several recent investigations. Lackovic et al. [18] studied the As removal kinetics from water in terms of removal efficiencies, but this type of kinetic information is linked to a concrete case and therefore not useful for determining general experimental conditions. The kinetics of As(V) removal by ZVI over a one-year period ranged between first- and zeroth-order regarding the aqueous As(V)concentration, and the reaction rate was dependent on the aqueous As(V) concentration and availability of adsorption sites. Kanel et al. [19] used a first-order model to describe As(V) removal by NZVI kinetics and illustrated iron’s capacity for As (V) removal using a Langmuir adsorption model. However, there has been no systematic study on the kinetics of As(V) elimination by nanoscale absorbent materials supported onto carriers. NZVI/AC can avoid the aggregation of NZVI, so it is easy to recover and reuse in engineering. Therefore, an in-depth understanding of its dynamics would help in accelerating the application of nanomaterials in the field of environmental remediation. However, the carrier also significantly increases its steric hindrance and decreases reaction opportunities, so it is necessary to organically study its kinetics, which are affected by many common factors.

We have previously [12] prepared activated carbon (AC)-supported NZVI by saturating AC with Fe^2+/3+^ followed by in-situ reduction to Fe^0^. In the present study, we investigated its characteristics, including the effects of adsorbent mass, initial As(V) concentration, pH of the solution, coexisting ions, and temperature on the reaction kinetics of As(V) removal, in a batch reaction system. The above parameters showed important effects on the dynamics of the adsorption system. The results are explained in terms of the intra-particle diffusion mechanisms. Furthermore, the kinetic model fitting the experimental data was used to analyze the performance of the NZVI/AC adsorption system in the removal of As(V) from raw (untreated) groundwater.

## 2. Experimental Section

### 2.1. Synthesis and Characterization of NZVI/AC

The NZVI/AC was synthesized as described elsewhere [12]. The morphology of the resulting samples was characterized by scanning electron microscopy (SEM) using the Hitachi SU 8220 instrument (SU8200, Hitachi, Tokyo, Japan). The surface area and porosity were calculated using the Brunauer–Emmett–Teller (BET) method from data including the N_2_ adsorption/desorption that were collected using a PMI BET-201A (Porous Materials Inc., Ithaca, NY, USA). The iron content was analyzed as described elsewhere [12]. Briefly, 0.1 g of adsorbent was added to 30 mL of 7 M HCl. The mixture was then placed into a water bath at 25 °C for 2 h, after which the temperature was slowly increased (over the next 20 min) to 90 °C. After the formation of a solid product, the supernatant was filtered and subjected to spectrophotometric analysis using an inductively coupled plasma optical emission spectrophotometer (ICP-OES; Varian 700-ES, Varian Inc. Scientific Instruments, Palo Alto, CA, USA).

### 2.2. Methods of Adsorption Kinetics

The effect of experimental parameters, including initial As(V) concentration (0.5, 1, 2, and 4 mg/L), adsorbent dosage (0.5, 1.0, 1.5, and 2.0 g/L), pH (3.5, 6.5, and 9.5), single and multiple coexisting ions (phosphate (${\mathrm{PO}}_{4}^{3-}$) and silicate (${\mathrm{SiO}}_{4}^{2-}$)) at a ratio to As(V) of 10:1, and temperature (298, 308, and 318 K), on the As(V) adsorption, was studied in conical flasks. For this purpose, 500 mL of an As(V) solution (2 mg/L) was added into each flask and the pH was adjusted to 6.5 using NaOH or HCl. The resulting mixture was shaken for 72 h at 298 K. Aliquots (5 mL) of the supernatants were filtered over a 0.45 µm Millipore filter. The As(V) content was then analyzed by hydride generation atomic fluorescence spectrophotometry (HG-AFS) using an AFS-2202E instrument (Haiguang Corporation, Beijing, China). The As(V) detection limit and analytic regression coefficient (R^2^) were 0.1 μg/L and >0.9990, respectively.

The multiple coexisting ions test was performed in the same way as the single coexisting ions test except for the presence of coexisting ions in the solution.

The residual concentration in the adsorbent (*q_t_*, mg/g) was calculated as follows

(1) $$q_{t}\;=\frac{V\left(C_{0}-C_{t}\right)}{W_{s}}$$

where *C*_0_ and *C_t_* are initial As(V) concentration (in mg/L) and the concentration of As(V) at time *t*, respectively; *V* is the solution volume (in L) and *W_s_* is the adsorbent weight (g). The percentage of removed As(V) (*R*%) was calculated by the equation shown below:

(2) $$R(\%)\;=\frac{C_{0}-C_{t}}{C_{0}}\times 100$$

### 2.3. Models

An intraparticle diffusion model [20] was applied to compute the As(V) adsorption kinetics in this study according to the equation

*q_t_* = *k_id_ t*^0.5^ (3)

where *k_id_* is the original rate of intraparticular diffusion (mg·g^−1^·h^−0.5^); *q_t_* (mg/g) is the quantity of As(V) adsorbed at time *t*.

### 2.4. The Qualitative Parameters of the Simulation and Raw Groundwater

The chemical composition of the simulation water included the specific ions context mentioned dissolved in deionized water.

To test the performance of our synthesized NZVI/AC in the removal of As(V) from raw groundwater, we used water from a pump well in Tumed Left Banner, Hohhot City, Inner Mongolia, a village affected by arsenicosis. The quality parameters of the raw water are listed in Table 1. The depth of the groundwater of the pump well was 6 m; water temperature: 13 °C; total hardness: 49.0 mg/L.

## 3. Results

### 3.1. Adsorbent Characterization

**Figure 1:**
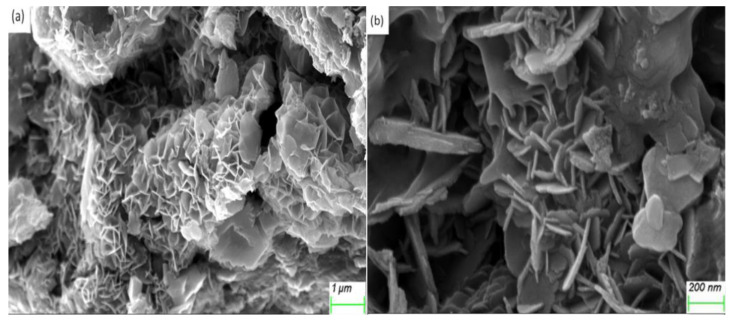
Scanning electron microscopy (SEM) images of the activated-charcoal (AC)—supported nanoscale zero-valent iron (NZVI) particles. (**a**) image at low magnification; (**b**) image at high magnification.

The SEM micrographs of NZVI/AC demonstrated that AC-supported NZVI particles of ~20 nm thickness were shaped like flakes (Figure 1). In comparison, clusters of round-shaped particle aggregates were formed by NZVI, which was synthesized in solution with a diameter <100 nm [7,11]. Most of the NZVI particles were located in the AC cracks and pores, which is an excellent benefit for future usage of this material for water treatment since it minimizes Fe^0^ nano-particle loss.

The Fe content of the NZVI/AC was 8.2%, whereas Fe was completely absent from unmodified AC. The total pore volume and BET surface area of the AC were 0.45 cm^3^/g and 821.7 m^2^/g, respectively, which decreased to 0.078 cm^3^/g and 69.4 m^2^/g, respectively, upon loading with NZVI, illustrating the remarkable changes to the porous structure of the AC upon loading with NZVI. These results were similar to those reported by Tan who studied the removal behavior of Se(IV)/Se(VI) from aqueous solution using biochar—and activated carbon-supported zerovalent iron, respectively [11].

### 3.2. Adsorption Kinetics of As(V) on NZVI/AC

#### 3.2.1. Effect of the Initial As(V) Concentration on Adsorption Kinetics

**Figure 2:**
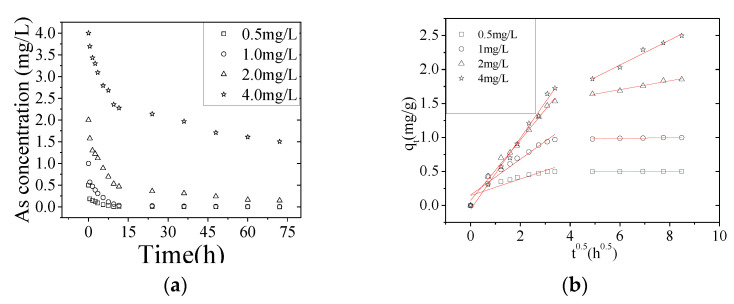
Adsorption kinetics of As(V) on NZVI/AC. (**a**) Effect of the initial concentration of As(V) on its adsorption on NZVI/AC as a function of incubation time. (**b**) Effect of the initial concentration of As(V) on q_t_ and intraparticle diffusion model fit for the As(V) removal at different initial As(V) concentrations by NZVI/AC. Conditions: pH = 6.5, 150 rpm, 20 × 40 mesh particle size, adsorbent dosage = 1.5 g/L, *t* = 72 h, T = 298 K, *C*_0_ = 0.5, 1.0, 2.0, and 4.0 mg/L.

The effect of the initial As(V) concentration on its adsorption on NZVI/AC was studied by incubation for 72 h at pH 6.5. When the initial As(V) concentration was changed from 0.5 to 4.0 mg/L the amount of removed As(V) decreased from 99.9 to 62.4% (Figure 2a) and the *q_t_* adsorption increased from 0.500 to 2.497 mg/g (Figure 2b). Thus, the removal of the As(V) was dependent on the original concentration. The same trend was observed for the adsorption of As(V) onto untreated powdered eggshell [21] and of Cr(VI) onto activated carbon stemming from wood apple shell [22].

The fast, initial adsorption gradually slowed down (Figure 2a). The adsorption equilibrium, reached within 72 h, was considered to be enough for the significant removal of As(V) and therefore 72 h was used for all the further experiments. Similar phenomena were seen with other initial concentrations.

To design the adsorption treatment systems, many models for liquid-phase adsorption were used to analyze the adsorption kinetic data but only the intraparticle diffusion model fitted well (R^2^ > 0.9) in this study.

Weber and Morris have illustrated that intraparticle diffusion is the rate-limiting factor in an adsorption system and the mass of the adsorbed substrate changes linearly as a function of the square root of time (*t*^1/2^). These data were then used to calculate the speeds of adsorption [20]. When *q_t_* was plotted as a function of *t*^1/2^, a linear relationship was seen in two separate stages (Figure 2b). Therefore, Equation (1) was applied to these two stages separately. The first linear section of this graph corresponded with the adsorption on the NZVI located inside the macropores while the second very likely corresponded with the diffusion of As(V) into micro- and/or mesopores. Adsorption of As(V) on the NZVI particles located inside the AC macropores or channels was quick, while their diffusion into micro- and mesopores was slow because most of these pores were blocked. Apart from the pore diffusion process, the corrosion of the ZVI surface, adsorption, and diffusion in the corrosion layers were also involved.

Two different *k_id_* values were calculated from the slopes of the two linear stages of the plots. The correlation coefficient (*R*^2^) values of the first and second stages of the plots at each *C*_0_ are listed in Table 2. All *k_id_* values of the 1st stage were higher than those of the 2nd stage, indicating a higher reaction velocity of the 1st stage compared with the 2nd stage, which was consistent with Figure 2b. Similar phenomena were found for adsorption of acid dye adsorption on activated palm ash [23] and Cu(II) and Cd(II) on rice/modified rice husk [24]. However, the rate constants, calculated *q_e_* values and were closer to the experimental *q_e_* values for the pseudo-second-order (PSO) model than for the pseudo-first-order model when using nanoscale zero-valent iron impregnated with clays adsorption and degradation of Zn^2+^ and Cu^2+^ from wastewaters. It can be concluded from the kinetic parameters that Cr(VI) removal by nZVI/BC is controlled by a chemical process, which indicates that adsorption/coprecipitation and reduction occurred during Cr(VI) removal. This is due to the fact that the clay has a too-low porosity rate [6]. Jingge Shang found that the PSO model provided a better fit with the kinetic data for removal of Cr(VI) by nanoscale zerovalent iron particles supported on herb-residue biochar. Therefore, the kinetic parameters are controlled by a chemical process, which indicates that adsorption/coprecipitation and reduction occurred during Cr(VI) removal [5].

#### 3.2.2. Effect of the Adsorbent Dosage on the Adsorption Kinetics

**Figure 3:**
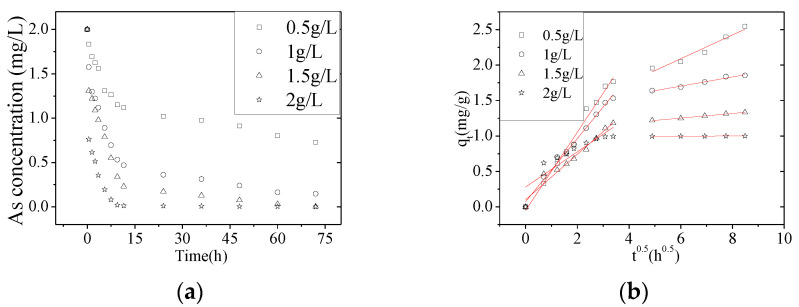
The As(V) removal rate as function of adsorbent concentration. (**a**) Effect of varying the adsorbent concentration on as function of time. (**b**) Intraparticle diffusion model fit for the As(V) removal rate by NZVI/AC at various adsorbent dosages (conditions: *C*_0_ = 2.0 mg/L, pH = 6.5, 150 rpm, 20 × 40 mesh particle size, *t* = 72 h, T = 298 K, adsorbent dosage: 0.5–2.0 g/L).

The As(V) removal rate increased from ~63.6 to ~100% when the NZVI/AC dose was increased from 0.5 to 2.0 g/L (Figure 3). However, the amount of adsorbed As(V) and the *k_id_*_1_ values decreased from 2.545 to 0.9990 mg/g and 0.5515 to 0.2482 mg·g^−1^·h^−0.5^, respectively (Table 3). This was due to the fact that as the mass of the adsorbent increased, the corresponding adsorption rate decreased because of the lower adsorption capacity of the adsorbent [19,23].

#### 3.2.3. Effect of pH on the Adsorption Kinetics

**Figure 4:**
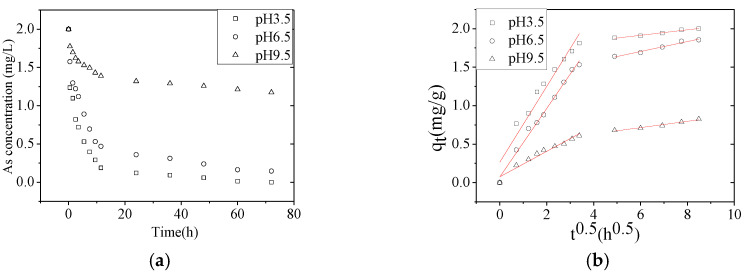
Effect of the pH on the As(V) removal rate. (**a**) Effect of the pH on the As(V) removal rate as a function of time. (**b**) Intraparticle diffusion model fit for the As (V) removal rate by NZVI/AC at different pH values (conditions: *C*_0_ = 2.0 mg/L, 150 rpm, 20 × 40 mesh particle size, *t* = 72 h, T = 298 K, adsorbent dosage = 1.5 g/L, pH = 3.5, 6.5, and 9.5).

Next, we tested the effect of the pH of the solution on the As(V) adsorption capacity of NZVI/AC. Our results showed that the removal rate of As(V) decreased with increasing pH value. The percent removal (*R*%) of As(V) by NZVI/AC reduced from 99.99% to 92.7% and 41.3% as the pH increased from 3.5 to 6.5 and 9.5, respectively (Figure 4).

The pH of the solution affects not only the speciation of heavy metals but also their ability to be removed using different types of functional groups and nanomaterials. The pH values will affect the ionization of acidic groups and the surface charges. Table 4 displays the values of calculated kinetic constants at pH 3.5, 6.5, and 9.5. All values of the correlation coefficients were >0.9362. The *k_id_*_1_ values decreased with increasing pH, indicating that the diffusion coefficients inside the particle pores decreased in the same pH range. As the pH increased from 3.5 to 6.5 and 9.5, the *k_id_*_1_ value for the As(V) adsorption on NZVI/AC decreased by 10.1% and 73.4%, respectively (Table 4).

Dissociation constants for the adsorption reactions performed at pH values of 3.5, 6.5, and 9.5 were equal to pK_a1_ = 2.3, pK_a2_ = 6.8, and pK_a3_ = 11.6 [24]. These differences occurred since, at these pH values As(V) exists as H_2_AsO_4_^−^, HAsO_4_^2−^, and AsO_4_^3−^ species, respectively. The pH of the zero-point charge (pH_ZPC_) of NZVI/AC is equal to 7.4. The adsorption of anionic species is most favorable at pH values below the pH_ZPC_ because then the adsorbent surface is positively charged. As the pH of the solution increases, the adsorbent surface becomes less positive, which decreases its attraction toward anionic As-species. The steric hindrance of H_2_AsO_4_^−^ is greater than that of HAsO_4_^2−^, which partially explains the lower *k_id_*_2(pH3.5)_ value compared with *k_id_*_2(pH6.5)_. Therefore, the adsorption of As(V) and its kinetics decreased significantly at higher pH values, which agrees with the previously reported literature on NZVI [19,25,26].

#### 3.2.4. Effect of Temperature on Adsorption Kinetics

**Figure 5:**
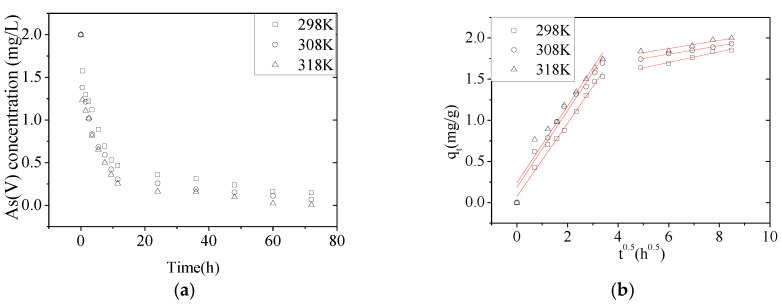
Effect of different temperatures on As(V) adsorption. (**a**) Effect of the temperature on the As(V) removal rate as a function of time. (**b**) Intraparticle diffusion model fit for the As(V) removal by NZVI/AC at different temperatures (conditions: *C*_0_ = 2.0 mg/L, pH = 6.5, 150 rpm, 20 × 40 mesh particle size, adsorbent dosage = 1.5 g/L, *t* = 72 h, T = 298, 308, 318 K).

The removal of the As(V) from solution by NZVI/AC was studied at 298, 308, and 318 K to determine the adsorption thermodynamic parameters (Figure 5). The *q_e_* of NZVI/AC increased from 1.854 to 1.998 mg/g upon raising the temperature of the As(V) solution from 298 to 308 K (Table 5). Similar trends were observed for the aqueous phase adsorptions [23,27].

The adsorption of As(V) onto NZVI/AC showed slower kinetics compared with NZVI. More than 80% of the As(V) was removed by NZVI within 7 min and ~99.9% was removed within 60 min [28]. As(V) adsorption on NZVI particles located inside AC macro-pores or -channels was slowed down by the carrier. However, NZVI possessing a tiny particle size displayed more As(V) adsorption due to its greater surface area.

Although the kinetics of As(V) adsorption by NZVI/AC was slower than by NZVI, loading NZVI with AC has several advantages, including (1) NZVI/AC avoids ferric loss, condensation, and further separation from the water system, thereby reducing water treatment costs; (2) NZVI/AC exhibits attrition resistance properties and excellent mechanical strength; and (3) NZVI/AC shows high recyclability and could sustain As(V) removal activity even after eight regeneration cycles. 

In summary, optimal conditions for the removal of As(V)from aqueous solutions were found to be 318 K, pH 3.5, 1.5 g/L adsorbent mass, and 72 h equilibrium time.

### 3.3. As(V) Adsorption in the Presence of Other Ions

#### 3.3.1. Effect of Single Coexisting Ions on Adsorption Kinetics

**Figure 6:**
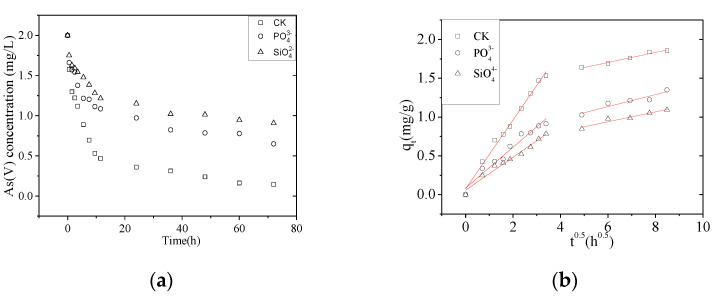
Effect of single interfering ions on As(V) adsorption. (**a**) Effect of single coexisting ions on As(V) removal as a function of time. (**b**) Intraparticle diffusion model fit for the As (V) removal by NZVI/AC in the presence of single coexisting ions (conditions: *C*_0_ = 2.0 mg/L, 150 rpm, 20 × 40 mesh particle size, *t* = 72 h, T = 298 K, adsorbent dosage = 1.5 g/L, pH = 6.5, coexisting ions = ${\mathrm{PO}}_{4}^{3-}$ or ${\mathrm{SiO}}_{4}^{2-}$). CK, control in the absence of interfering ions.

Drinking water sources are likely to contain several ions that might compete with As for the applicable adsorption sites on NZVI/AC. The effects of coexisting ions on As(V) removal depend on their type and concentration. Here, we probed the effects of ${\mathrm{PO}}_{4}^{3-}$ and ${\mathrm{SiO}}_{4}^{2-}$ as interfering ions because they are frequently found in groundwater streams and might either inhibit or enhance As(V) removal kinetics (Figure 6).

Several studies have shown that ${\mathrm{PO}}_{4}^{3-}$ and ${\mathrm{SiO}}_{4}^{2-}$ inhibit As(V) removal [19,25,26,28,29]. In our study, the *k_id_*_1_ of *k*_p_ and *k*_si_ decreased to 59.2% and 47.7%, respectively, compared with the control (Table 6). The As(V), ${\mathrm{PO}}_{4}^{3-},\;$and ${\mathrm{SiO}}_{4}^{2-}$ are all tetrahedral anions and generate inner-sphere complexes with the effective functional groups at the surface of iron oxides. Competition for adsorption sites reduces the adsorption of either negative ion when both are present compared with either single anion. Likewise, ZNVI/AC acted as a positive adsorbent for both ${\mathrm{PO}}_{4}^{3-}$ and ${\mathrm{SiO}}_{4}^{2-}$ and the presence of either one in excess concentration over As(V) in groundwater would cause imperfect removal of As(V). Alternatively, an excess of ZNVI/AC may be used to assure the complete removal of As(V) in the presence of strong competing anions [30].

#### 3.3.2. Multiple Coexisting Ion Effect on Adsorption Kinetics

The above results described the effect of single commonly coexisting ions on the adsorption kinetics of As(V). However, groundwater contains many coexisting ions simultaneously.

**Figure 7:**
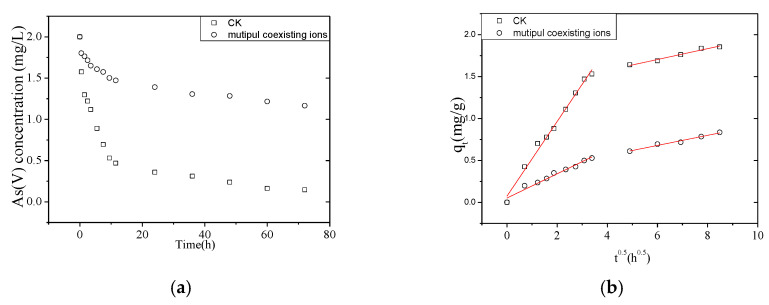
Effect of multiple interfering ions on As(V) adsorption. (**a**) Effect of multiple coexisting ions on the removal of As(V) as a function of time. (**b**) Intraparticle diffusion model fit for the removal of As(V) by NZVI/AC in the presence of multiple interfering ions (conditions: *C*_0_ = 2.0 mg/L, 150 rpm, 20 × 40 mesh particle size, *t* = 72 h, T = 298 K, adsorbent dosage = 1.5 g/L, pH = 7.58 (i.e., natural pH), coexisting ions = ${\mathrm{PO}}_{4}^{3-}$, ${\mathrm{SiO}}_{4}^{2-}$).

**Figure 8:**
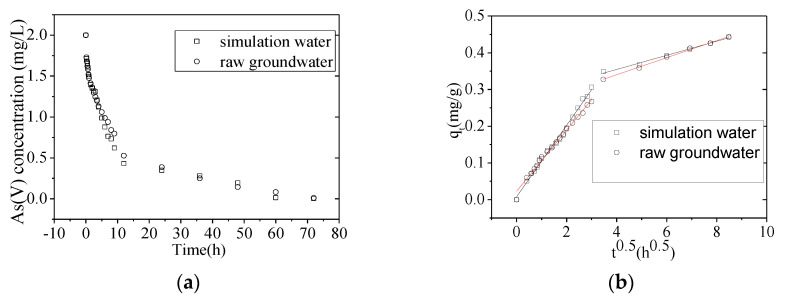
Adsorption of As(V) from raw groundwater. (**a**) Performance of NZVI/AC in the removal of As(V) from raw groundwater compared with water containing only As(V). (**b**) Intraparticle diffusion model fit for the As (V) removal by NZVI/AC in raw groundwater compared to simulation water containing only As(V) (conditions: *C*_0_ = 2.0 mg/L, 150 rpm, 20 × 40 mesh particle size, *t* = 72 h, T = 298 K, adsorbent dosage = 4.5 g/L, pH = 6.5).

Therefore, we next studied the effect of multiple simultaneously coexisting ions and raw groundwater on As(V) removal (The raw ground-water quality parameters see Table 1). The As(V) removal rate in simulated groundwater containing multiple coexisting ions was only 41.7%, while, in the control check water (containing only As(V)) the removal rate was 92.7% (Figure 7). In order to meet the Chinese drinking water standard (GB 5794-2006: As(V) ≤ 0.01 mg/L), the adsorbent dosage needed a two-fold increase (4.5 g/mL) for purifying raw groundwater. As(V) removal rate from the simulated water and raw groundwater was ≥99.9% in both cases (Figure 8).

The adsorption kinetics of As(V) in the simulated groundwater containing multiple coexisting ions was slower than in water containing single coexisting ions (Table 7 and Table 8).

As(V) was adsorbed as an inner sphere complex on iron oxyhydroxides which corroded from NZVI/AC in other previous studies. Phosphorus and arsenic belong to VA and have similar atomic structures and chemical properties, sharing the same outer shell of s^2^p^3^ [26]. Both phosphate and silicate bonded with iron hydroxides and competed for adsorption sites with arsenic. They would compete for similar adsorptive binding sites, hence decreasing the removal rate of arsenic [13]. The reduction in available adsorption sites would weaken the adsorption rate and adsorption capacity of NZVI/AC [19]. This may be attributed to a decrease in adsorbent pores, surface area, the number of unsaturated sites, and active sites [31]. In addition, the radius of P/As anions is similar, which respectively 0.248, 0.238 nm. Isomorphous replacement occurs when different ions yield slightly soluble crystals in a similar geometric form. Therefore, the ${\mathrm{PO}}_{4}^{3-}$ substitution of ${\mathrm{AsO}}_{4}^{3-}$ in the NZVI/AC, which decreased the adsorption kinetics, too [32].

## 4. Conclusions

The adsorption kinetics *k_id_*_1_ of the As(V) increased from 0.1224 to 0.5251 with increasing initial As(V) concentration from 0.5 to 4.0 mg/L. However, the *k_id_*_1_ decreases from 0.4921 to 0.1654 when the pH increases from 3.5 to 9.5, and when the adsorbent dosages increase from 0.5 to 2.0 g/L, the *k_id_*_1_ decreases from 0.5515 to 0.2482. The kinetics of the As(V) adsorption to NZVI/AC in the simulated groundwater containing single or multiple coexisting ions was slower by about 1 and 3 times than for the simulated groundwater without coexisting ions. We found that intraparticle diffusion exhibited the rate-determining effects on the As(V) removal process which resulted from the relatively simple macropore structure of the NZVI/AC. The results of the present investigation indicate that NZVI/AC is a promising As(V) remover. The NZVI/AC adsorbent does not share the disadvantages of other nano-iron particles that readily agglomerate, and NZVI/AC is easily separated from the water. Further work is required to determine if NZVI/AC can be applied for the removal of other harmful components in water.

## Figures and Tables

**Table 1:** The raw ground-water quality parameters.

As (mg/L)	pH	TOC (mg/L)	NH_3_-N (mg/L)	Fe (mg/L)	SO_4_^2−^ (mg/L)	Cl^−^ (mg/L)	F^−^ (mg/L)	Na^+^ (mg/L)	K^+^ (mg/L)	Al^3+^ (mg/L)
1.971	7.58	12.3	0.475	4.6	349	174	1.62	137	3.9	1.04

**Table 2:** Rate constants for the kinetic model of the adsorption of As(V) on NZVI/AC at different initial As(V) concentrations.

Parameter	Weber–Morris Diffusion			
	1st Step		2nd Step	
*C*_0_ (mg/L)	*k_id_*_1_	R^2^	*k_id_*_2_	R^2^
0.5	0.1224	0.7636	0.0004	0.9774
1.0	0.2632	0.9293	0.0060	0.9341
2.0	0.4449	0.9900	0.0648	0.9673
4.0	0.5251	0.9913	0.1835	0.9769

**Table 3:** Rate constants for the kinetic model of the adsorption of As(V) on NZVI/AC at different adsorbent dosages.

Parameter	Weber–Morris Diffusion			
	1st Step		2nd Step	
*C*_0_ (mg/L)	*k_id_*_1_	R^2^	*k_id_*_2_	R^2^
0.5	0.5515	0.9838	0.1677	0.9399
1.0	0.4449	0.9900	0.0647	0.9672
1.5	0.3203	0.9662	0.0326	0.9934
2.0	0.2482	0.7790	0.0012	0.9234

**Table 4:** Rate constants for the adsorption kinetic model of As(V) on NZVI/AC at different pH values.

Parameter	Weber–Morris Diffusion			
	1st Step		2nd Step	
pH	*k_id_*_1_	R^2^	*k_id_*_2_	R^2^
3.5	0.4921	0.9362	0.0355	0.9731
6.5	0.4449	0.9899	0.0647	0.9673
9.5	0.1654	0.9532	0.0409	0.9646

**Table 5:** Rate constants for the kinetic model of As(V) adsorption on NZVI/AC at different temperatures.

Parameter	Weber–Morris Diffusion			
	1st Step		2nd Step	
T(K)	*k_id_*_1_	R^2^	*k_id_*_2_	R^2^
298	0.4449	0.9900	0.0647	0.9673
308	0.4657	0.9677	0.0203	0.9926
318	0.4664	0.9503	0.0283	0.8860

**Table 6:** Rate constants for the kinetic model of As(V) adsorption on NZVI/AC in the presence of single coexisting ions.

Parameter	Weber–Morris Diffusion			
	1st Step		2nd Step	
Single Coexisting Ions	*k_id_*_1_	R^2^	*k_id_*_2_	R^2^
CK ^1^	0.4449	0.9900	0.0647	0.9673
${\mathrm{PO}}_{4}^{3-}$	0.2633	0.9577	0.0776	0.8741
${\mathrm{SiO}}_{4}^{2-}$	0.2123	0.9756	0.0638	0.9180

^1^ CK = control check.

**Table 7:** The adsorption kinetic model rate constants for the As (V) on NZVI/AC in the presence of multiple coexisting ions.

Parameter	Weber–Morris Diffusion			
	1st Step		2nd Step	
Multiple Coexisting Ions	*k_id_*_1_	R^2^	*k_id_*_2_	R^2^
CK	0.4449	0.9900	0.0648	0.9673
multiple coexisting ions	0.1447	0.9679	0.0602	0.9722

**Table 8:** Rate constants for the kinetic model of As(V) adsorption on NZVI/AC. Comparison of simulation water containing only As(V) vs. raw groundwater.

Parameter	Weber–Morris Diffusion			
	1st Step		2nd Step	
	*k_id_*_1_	*R* ^2^	*k_id_*_2_	*R* ^2^
simulation water	0.09725	0.9922	0.01922	0.9907
raw groundwater	0.0832	0.9902	0.02323	0.9952
